# The Effect of Particle–Matrix Interface on the Local Mechanical Properties of Filled Polymer Composites: Simulations and Theoretical Analysis

**DOI:** 10.3390/polym17010111

**Published:** 2025-01-03

**Authors:** Timur A. Nadzharyan, Elena Yu. Kramarenko

**Affiliations:** 1 Faculty of Physics, Lomonosov Moscow State University, Moscow 119991, Russia; nadz@polly.phys.msu.ru; 2 Enikolopov Institute of Synthetic Polymeric Materials, Russian Academy of Sciences, Moscow 117393, Russia

**Keywords:** polymer composites, stiffening effect, anisometric particles, particle–matrix interface, filler–matrix interface, interphase, theoretical modeling, magnetoactive elastomers

## Abstract

A finite element model of the local mechanical response of a filled polymer composite to uniaxial compression is presented. The interfacial layer between filler particles and polymer matrix is explicitly modeled as a third phase of the composite. Unit cells containing one or several anisometric filler particles surrounded by interface shells are considered. The dependence of the mechanical response of the cells to external deformation on the interface thickness and stiffness is studied. The use of the particle–matrix interface as a damping tool in mesoscopic polymer-composite problems with large deformations is discussed. The influence of the interface on the anisotropy of the composite response is considered.

## 1. Introduction

Filled polymer composites are materials made by combining a polymer matrix with filler particles that are dispersed throughout the matrix. The polymer acts as the dispersion phase, providing the overall structure and binding the filler particles together. The different types of fillers are solids, fibers, and particulates, and they are added to enhance or modify the properties of the polymer, such as its strength, stiffness, thermal conductivity, electromagnetic properties, or durability [1,2,3,4,5,6]. By integrating different types of fillers or changing the filler concentration and distribution, these composites can be tailored for a wide range of industrial applications, offering improved performance and cost-effectiveness. A special group of such materials is magnetosensitive composites with a soft polymer matrix or magnetoactive elastomers (MAEs) [7,8,9,10,11,12,13]. The sensitivity to magnetic field is achieved by adding ferromagnetic or ferrimagnetic particles to the composite. These particles are usually micron- or nanometer-sized and can move within the composite under the influence of external magnetic fields. When an external magnetic field is applied, the magnetic particles within the elastomer align along the field lines, often forming chain-like structures. This rearrangement of the particles causes changes in the mechanical properties of the material: increased material stiffness [12,13,14], induced volumetric deformation [15,16] and changes in surface roughness [17,18,19,20,21,22,23]. The ability to control and modify physical properties in real time leads to MAEs being referred to as “smart materials” and makes them highly versatile and useful for a variety of applications [24,25,26,27,28,29,30].

Different types of responses that magnetic fields can induce in MAEs include the magnetorheological effect or the dependence of the viscoelastic properties of the composite on the external magnetic field [8,10,12,13,31]; changes in the shape and dimensions of the material sample in the presence of both uniform and non-uniform magnetic fields or magnetodeformation effects [32,33,34,35]; and changes in electromagnetic properties of the material, i.e., the dielectric permittivity [36,37], magnetic permeability [38,39], magnetization curves [40,41], and conductivity [42,43]. In many cases, these changes are significant but reversible, making MAEs very flexible. Magnetomechanical coupling significantly influences all of these effects, and therefore both the mechanical and magnetic properties of the composite components merit detailed study in order to fine-tune the characteristics of the resulting composite.

The wide variety of ways magnetic field can influence the structure and behavior of MAEs, as well as the possible ranges of their physical properties, creates many possible practical applications of such materials. Notable applications include the usage of MAEs in civil and mechanical engineering as parts of vibration dampers and isolators to control vibrations in buildings, bridges, or machinery [7,44,45,46,47]; in sensors that detect magnetic fields or pressure, and as actuators for controlled motion [26,48,49,50,51,52]; as parts of biomedical devices, such as wearable devices, adaptive compression systems, and tunable medical seals [53,54,55,56,57,58]; as coatings with adjustable roughness, adhesion, or wettability [59,60,61,62]; as frequency filters for communication systems [63,64,65]; and as actuators that enable controlled motion and flexibility in soft robotics [66,67,68,69,70]. The complexity of the physical phenomena exhibited by MAEs and the diversity of their practical applications are the main reasons for the very active interest of many researchers in these smart materials.

A key challenge in understanding and utilizing filled polymer composites lies in theoretical modeling that is essential for optimizing their practical applications. The complexity of a consistent theoretical approach to describing composite materials stems from the need to solve coupled physical problems (i.e., magnetomechanical problems) and to consider processes occurring at different scales (mesoscopic scale, corresponding to the filler particles; and macroscopic scale, corresponding to the entire material sample). Due to the complex material behavior resulting from filler particle rearrangement and the polymer matrix response, various theoretical modeling approaches have been developed over the years to explain the properties of polymer composites. One of the more general approaches is continuum modeling, which treats the material as a whole, without focusing on the detailed behavior of individual filler particles. Continuum models are often used to describe macroscopic behavior, such as bulk deformation or the overall thermal, magnetic, and dielectric response of the material [71,72,73]. An invariant-based analytical framework, originally developed for dielectric elastomers, has also been applied to MAEs, focusing on materials with isotropic responses [74,75]. In this approach, the material is considered as a homogeneous system, and its properties are averaged out, resulting in less resource-heavy calculations. However, continuum models often fail to capture the fine details of filler particle restructuring, a critical component of composite behavior. Microscopic and mesoscopic models address this aspect of the material behavior. These models explicitly account for the presence and interactions of individual filler particles within the polymer matrix. In microscopic models, the position, orientation, and properties of each filler particle or particle aggregate are considered with respect to both the applied external fields and the surrounding particles [76,77,78,79,80]. This allows for a more detailed understanding of how particle interactions affect both the local and macroscopic properties of the material. The complexity of polymer composite materials, involving both macroscopic and microscopic phenomena, has led to the development of multi-scale models [81,82,83,84,85]. These models aim to bridge the gap between the microscopic behavior of individual filler particles and the overall macroscopic response of the material. Multi-scale approaches incorporate elements from both continuum and microscopic models, allowing for a more comprehensive understanding of composite materials, especially MAEs and dielectric elastomers, under various conditions, such as different external field strengths, particle concentrations, and matrix stiffness.

In the field of MAE studies, most theoretical models to date assume that filler particles have a spherical shape. However, more recent research has explored the effects of anisotropic (non-spherical) particles, such as rods or platelets, on the material behavior [86,87,88,89]. The shape of particles can significantly influence the material’s magnetic response and deformation. MAEs with anisotropic fillers exhibit direction-dependent behavior, as well as a significantly higher magnetorheological effect at low filler concentrations, adding complexity to models that typically assume isotropic materials.

Another aspect of the material structure that is not often considered in theoretical studies is the interface region between the filler particles and the polymer matrix (interphase). Interactions of the polymer chains with the particle surface can lead to the formation of either depletion or adsorption layers with properties different from the bulk of the material. In Ref. [90], where the role of the polymer–magnetic nanoparticle interface was studied via reactive dissipative particle dynamics, it was found that the interface contributes to the tensile stiffness of composites. In addition, particle surface modification is often used in the synthesis of polymer composites to improve particle–polymer compatibility and to achieve a more homogeneous distribution of fillers within the polymer matrix. The grafting of polymer chains onto the particle surface results in the formation of a protective layer compatible with the matrix [8,91,92,93,94,95,96,97,98]. Coatings for micron-sized particles with high thickness were obtained and studied in [99,100]. A general overview of the influence of the filler particle–matrix interface on the properties of polymer composites can be found in [101,102].

The surface layer that surrounds a filler particle serves as an interface between the polymer matrix and filler and can both increase and decrease the resulting elastic modulus of the material depending on its properties. Thus, it can be used to fine-tune the mechanical characteristics of the material. This aspect of the composite design has been studied theoretically using molecular dynamics simulations [103] and multi-phase models [104]. Additionally, the particle–matrix interface can be a useful modeling tool that increases the stability of finite-element calculations for problems that involve significant filler restructuring and large deformations. Two-dimensional simulations of a mesoscopic element of a MAE containing circular filler inclusions surrounded by particle–matrix interface were presented in [105]. The research presented in this work focuses on 3D simulations and anisometric filler particles. The proposed approach allows for explicit consideration of the interfacial region between the filler particles and polymer matrix corresponding to either adsorption layers formed on the particle surface or particle coatings. Using mesoscopic FEM simulations, it is possible to calculate how geometric and physical parameters of the interface affect the bulk properties of the composite containing not only spherical particles, but also particles with an arbitrary isometric anisometric shape. The model itself is scalable and can be applied to soft and stiff composites, nanocomposites, and polymer materials containing micron-sized fillers.

This paper is structured as follows: The general modeling setup is explained in Section 2. In Section 3, cells containing a single filler particle surrounded by an interface shell are considered, their mechanical response is calculated and presented. A potential use of particle–matrix interface as a stabilization tool for mesoscopic continuum problems with large deformation is analyzed in Section 4. Cells containing several filler particles with the particle–matrix interface surrounding them are then considered in Section 5. The influence of the interface on the anisotropic mechanical response of the unit cell is evaluated. Finally, the concluding remarks are presented in Section 6.

## 2. Modeling Outline

**Figure 1:**
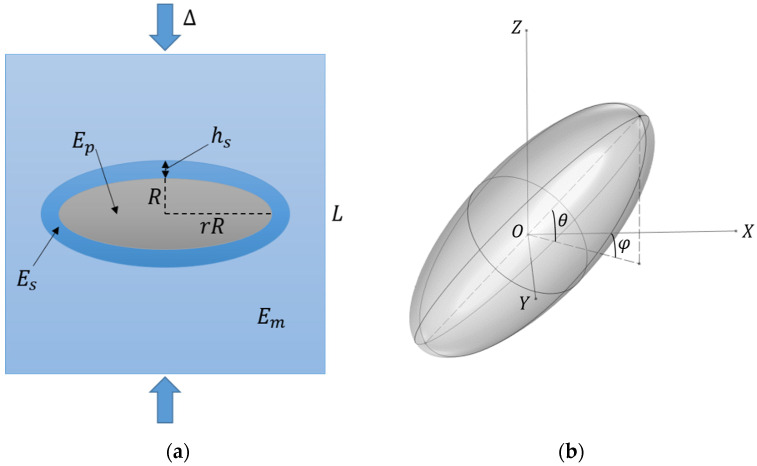
(**a**) A simple setup of a single-particle cell containing an ellipsoidal particle of radius (R) and the anisometry ratio (r) and an interface of the thickness ($h_{s}$) between the particle and the polymer matrix. Arrows indicate a uniaxial compression of the cell. (**b**) The spatial orientation of a prolate ellipsoidal particle at the center of the cell is characterized by two angles, θ and φ.

The basic modeling setup is presented using a single-particle cubic cell for the sake of visual clarity (Figure 1). Particles are assumed to be micron-sized, and the volume concentration, *c*, defines the linear size of the cell, L. The cell is compressed with the Cauchy strain parameter, ∆. The unit cell that represents the mesoscopic behavior of the material contains three phases: polymer matrix, filler particles, and particle–matrix interface surrounding the particles. Each phase is characterized by its mechanical properties, namely Young’s modulus (E) and Poisson’s ratio (ν). Let us denote the Young’s modulus and the Poisson’s ratio of the polymer matrix as $E_{m}$ and $\nu_{m}$, the Young’s modulus and the Poisson’s ratio of the filler particles as $E_{p}$ and $\nu_{p}$, and the Young’s modulus and the Poisson’s ratio of the shell surrounding the particles as $E_{s}$ and $\nu_{s}$, respectively. In this work, filler particles of spherical and ellipsoidal shapes are considered. As such, the filler phase is characterized by the linear size, R, which corresponds to the particle radius or the length of its small semi-axis; and the anisometry ratio, r, or the ratio of the major axis length to minor axis length. For spherical particles, r=1. The interface-phase dimensions are characterized by the interface thickness, $h_{s}$. The spatial orientation of the anisometric particles is characterized by two angles, θ and φ (see Figure 1b).

Both the cell medium and the shell are assumed to consist of nearly incompressible hyperelastic materials to simulate the properties of polymer materials. The deformation of the cell is assumed to be caused by neighboring cells and particle movement inside them. The strain energy density in this case can be expressed as a sum of the isochoric neo-Hookean and volume-changing Hartmann–Neff terms [106]:

(1) $$\Psi_{h}\left(I_{1},J\right)=\frac{G}{2}\left(J^{-2/3}I_{1}-3\right)+\frac{K}{50}\left(J^{5}+J^{-5}-2\right),$$

where G and K are the shear and the bulk moduli of the material, J=detF, $I_{1}=\mathrm{tr}C$, F is the deformation gradient tensor, and $C=F^{T}F$ is the right Cauchy–Green deformation tensor. The Young’s modulus of the filler particles is assumed to be significantly higher than the moduli of the polymer matrix and the shell, so their mechanical behavior can be described using linear theory of elasticity:

(2) $$\Psi_{l}\left(\varepsilon \right)=G\mathrm{tr}\left(\varepsilon^{2}\right)+\frac{\lambda }{2}{\mathrm{tr}}^{2}\varepsilon ,$$

where ε is the Cauchy strain tensor, and λ is the second Lame parameter. If the piecewise strain energy density function that combines the expressions for energy in different materials is denoted as Ψ, then the total energy stored in the cell can be calculated as follows:

(3) $$W_{c}=\int_{Vc}\Psi dV,$$

where $V_{c}$ is the volume of the unit cell. For the case of a cubic cell, it is equal to $L^{3}$. Let us denote the volume of a single filler particle as $V_{p}$ and the volume of the shell as $V_{s}$. The Young’s modulus of the cell can be calculated using the following relation:

(4) $$E_{c}=\frac{2W_{c}}{V_{c}{∆}^{2}},$$

The ferromagnetic filler is assumed to have mechanical properties of carbonyl iron. Our calculations show that the dependence, $E_{c}\left(E_{p}\right)$, reaches saturation rather quickly when $E_{p}>E_{m}$, so for relatively soft polymer matrices ($E_{m}<100$ kPa), the choice of filler does not noticeably impact mechanical characteristics of the cell as $E_{p}\gg E_{m}$ for all widely used fillers, and the results obtained for carbonyl iron can be used to describe the mechanical behavior of the cell containing filler particles made of different ferromagnetic materials.

Commercially available FEM software COMSOL Multiphysics 6.0 was used for simulations. The mechanical boundary conditions for the unit cell provided prescribed displacement at the boundary corresponding to ∆=0.1% of L. Low external mechanical load represents the filler particles sparsely distributed in the composite or low filler concentration. Adaptive mesh refinement was employed with the refinement process termination determined by the relative change in $E_{c}$. The mesh refinement process was terminated upon reaching the desired relative tolerance of 1%.

## 3. Unit Cell Containing a Single Particle with a Shell

In order to describe the influence of a shell surrounding a filler particle on the mechanical properties of the composite, first the simplest case of cells containing a single filler particle is studied.

**Figure 2:**
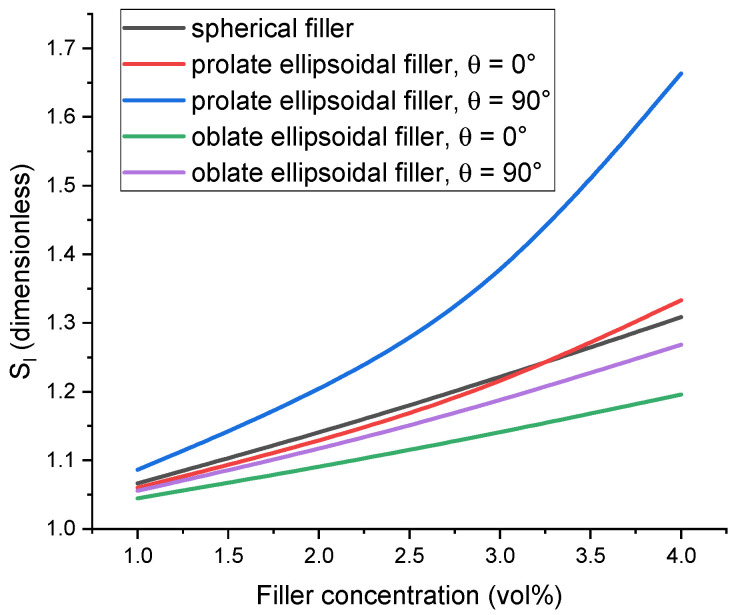
The dependences of $S_{l}=E_{c}\left({\tilde{E}}_{s}=100\right)/E_{c}\left({\tilde{E}}_{s}=0.01\right)$ on filler volume concentration that showcase how changing the stiffness of the interface can affect the elastic modulus of the cell. Filler particle anisometry is set at r=3, and the interface thickness is set at ${\tilde{h}}_{s}=0.2$.

As a preliminary justification of the importance of this issue, the extent to which the mechanical response of the cell can be affected by introducing a shell surrounding filler particles into the system is evaluated. To quantify this, the difference between the elastic modulus of a cell in the case of a stiff interface (at the saturation level of reinforcement) and the case of a soft interface (while maintaining computation stability) is considered. Let us then denote ${\tilde{E}}_{c}=E_{c}/E_{m}$ as the normalized cell modulus and ${\tilde{E}}_{s}=E_{s}/E_{m}$ as the normalized interface modulus. Figure 2 demonstrates how the ratio $S_{l}$ of ${\tilde{E}}_{c}$ corresponding to ${\tilde{E}}_{s}=100$ to ${\tilde{E}}_{c}$ corresponding to ${\tilde{E}}_{s}=0.01$ changes with filler volume concentration for filler particles of different shapes and spatial orientations and normalized interface thickness, ${\tilde{h}}_{s}=h_{s}/R$ of 0.2. At a filler concentration of about 5%, these conditions lead to a difference between the stiff and soft interface cases of about 30% or more depending on the filler shape and structure. The response of the cells containing prolate ellipsoidal filler particles arranged in chain-like structures demonstrates the highest degree of possible tuning using the particle–matrix interface, but even for the case of spherical particles, the influence of the interface is noticeable. It can also be seen that both the modulus ${\tilde{E}}_{c}$ and the ratio $S_{l}$ are noticeably higher for unit cells containing prolate ellipsoidal particles with θ=90°, which, according to our modeling setup (see Figure 1), corresponds to filler structuring in the direction of the mechanical load. In our previous work [89], it was found that this configuration leads to the highest value of the cell modulus. Specifically, for the case of prolate ellipsoidal particles, the minimum distance between the particle and the cell boundary decreases rapidly with increasing filler concentration and decreases even further due to the presence of the particle–matrix interface, which causes boundary effects to become significant. Because of that, the ${\tilde{E}}_{c}={\tilde{E}}_{c}\left(c\right)$ and ${\tilde{E}}_{c}={\tilde{E}}_{c}\left(r\right)$ dependences demonstrate an unrealistically sharp increase with increasing c and r, respectively, in this case, so the reliability of the model used in this work becomes questionable. As such, results obtained under these conditions should be analyzed as general trends and not as quantitatively accurate representations of the processes occurring in polymer composites.

**Figure 3:**
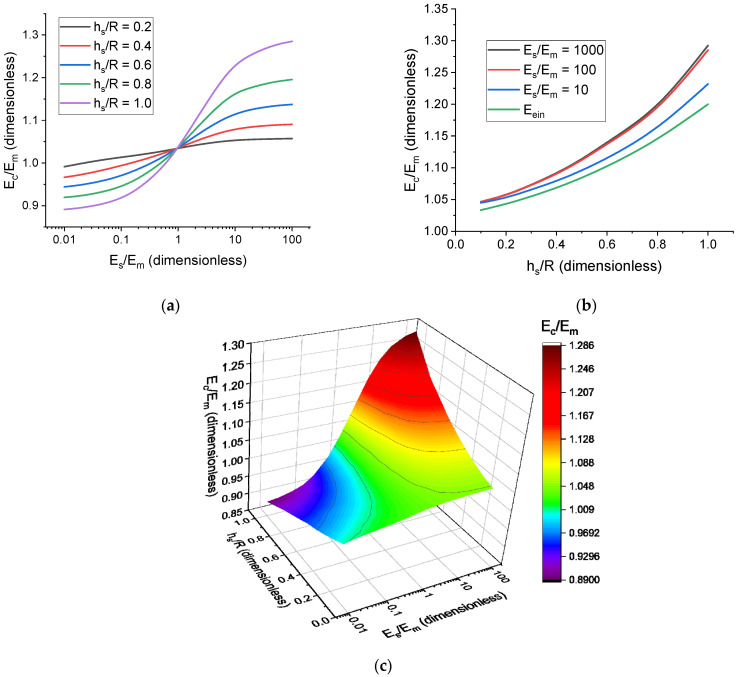
The dependences of the normalized cell modulus, ${\tilde{E}}_{c}$, on the normalized interface modulus, ${\tilde{E}}_{s}$ (logarithmic scale), and the normalized interface thickness, ${\tilde{h}}_{s}$. The presented dependences correspond to spherical particles and filler volume concentration of 1%. (**a**) Dependences of ${\tilde{E}}_{c}$ on ${\tilde{E}}_{s}$ (logarithmic scale) for different interface thickness values, ${\tilde{h}}_{s}$. (**b**) Dependences of ${\tilde{E}}_{c}$ on ${\tilde{h}}_{s}$ for different interface modulus values, ${\tilde{E}}_{s}$, and comparison with the results provided by Einstein’s formula, $E_{ein}$ [107], with increased effective filler concentration. (**c**) The combined dependence of ${\tilde{E}}_{c}$ on ${\tilde{E}}_{s}$ (logarithmic scale) and ${\tilde{h}}_{s}$.

Figure 3 demonstrates the dependences of the normalized cell modulus, ${\tilde{E}}_{c}$, on the normalized interface thickness, ${\tilde{h}}_{s}$, and normalized interface modulus, ${\tilde{E}}_{s}$. The dependence of ${\tilde{E}}_{c}$ on ${\tilde{E}}_{s}$ has the shape of a sigmoid function with saturation corresponding to $E_{s}$ reaching the value of the Young’s modulus of the particle $E_{p}$ (Figure 3a). This saturation, however, is reached at much lower values of $E_{s}$, especially in the case of a moderately thick interface (${\tilde{h}}_{s}\leq 0.5$), and ${\tilde{E}}_{s}\sim 100$ can be considered to effectively satisfy the saturation conditions; therefore, the presence of the interface increases the filler volume concentration from $V_{p}/V_{c}$ to $\left(V_{p}+V_{s}\right)/V_{c}$, which is denoted as an effective filler concentration, $c_{eff}$. This, in turn, increases $E_{c}$ by $\sim 2.5E_{m}V_{s}/V_{c}$ (following Einstein’s formula for filler reinforcement [107]) for low filler concentration values. Quick saturation of ${\tilde{E}}_{c}$ with ${\tilde{E}}_{s}$ allows for reaching the desirable material-stiffening effect without the need for excessively large interfacial region. If ${\tilde{E}}_{s}<1$, then, as one would expect, ${\tilde{E}}_{c}$ falls below the value of the modulus corresponding to the system without particle–matrix interface. However, as ${\tilde{E}}_{s}$ is decreased further, a critical value ${\tilde{E}}_{s}^{cr}$ can be reached that corresponds to the interface with very low Young’s modulus and the mechanical response of the cell becoming weaker than that of the pure polymer matrix. The simulations become less stable for ${\tilde{E}}_{s}<{\tilde{E}}_{s}^{cr}$, and material rupturing can occur. As such, this case will not be studied in detail in this work. Due to the considerations presented above, the mechanical behavior of the unit cells is studied for $0.01\leq {\tilde{E}}_{s}\leq 100$. This range of ${\tilde{E}}_{s}$ values is sufficient for both studying the saturation behavior of ${\tilde{E}}_{c}\left({\tilde{E}}_{s}\right)$ and the softening of the unit cell when ${\tilde{E}}_{s}<1$, while maintaining simulation stability.

The dependence of ${\tilde{E}}_{c}$ on ${\tilde{h}}_{s}$ is also nonlinear (Figure 3b) and can be analyzed within the framework of additional effective filler concentration. Comparing the obtained results with theoretical values given by Einstein’s model (or Guth–Gold model) [107,108] with effective volume concentration shows that this approach can indeed serve as a framework for studying interface-related problems. The shape of the ${\tilde{E}}_{c}\left({\tilde{h}}_{s}\right)$ is either polynomial or exponential, which is in agreement with filler reinforcement theory. The dependence of ${\tilde{E}}_{c}$ on both ${\tilde{E}}_{s}$ and ${\tilde{h}}_{s}$ is shown in Figure 3c.

A notable point of consideration is the value of $E_{c}$ for two special cases: when the mechanical properties of the interface are the same as the mechanical properties of the polymer matrix and when the mechanical properties of the interface are the same as those of the filler particle. It is reasonable to assume that the first case is practically identical to no interface being present (at least within the continuum modeling approach), and the second case corresponds to a larger filler particle with the volume of $V_{p}+V_{s}$ and the combined dimensions of the particle with the interface. Taking that into account, it is proposed to model the interface between the matrix and the filler as additional filler concentration, ∆c, that forms effective filler volume concentration, c+∆c, when ${\tilde{E}}_{s}\geq 1$. This assumption will lead to exact expressions for the elastic modulus only for the aforementioned two cases, but it could be a useful tool as a first-order approximation of the mechanical properties of the cell.

Classic filler reinforcement theory [109,110] provides an exponential model for the shear modulus of the composite filled with anisometric particles:

(5) $$\frac{G_{c}}{G_{m}}=\exp\left(\frac{2.5+0.407{\left(r-1\right)}^{1.508}}{1-Pc}c\right),$$

where P is the crowding factor representing the hydrodynamic interaction between filler particles that prevents close packing. This factor depends on the particle shape and polydispersity and is predicted to range from 1.35 to 1.91. Rewriting Expression (5) for the Young’s modulus, $E_{c}$, expanding it for low concentration values, and keeping only the first-order terms, one can obtain a simple general expression:

(6) $$\frac{E_{c}}{E_{m}}\approx 1+k_{1}c,$$

The first expansion coefficient, $k_{1}$, depends on $V_{c}$, r, and P. Now, assuming the presence of the interface with thickness, $h_{s}$, between the matrix and the filler, Expression (6) can be modified:

(7) $$\frac{E_{c}}{E_{m}}\approx 1+k_{1}c+k_{1}∆c\cdot S\left({\tilde{E}}_{s}\right),$$

Here, the additional effective concentration is $∆c=∆c\left(h_{s}\right)$, and $S\left({\tilde{E}}_{s}\right)$ is a saturation-type function that can be seen in Figure 3a. The case of $E_{s}=E_{m}$ corresponds to ∆c=0, as discussed above, so $k_{1}$ can be expressed in a simple manner:

(8) $$k_{1}=\frac{E_{0}-1}{c},$$

where $E_{0}=E_{0}\left(c,r\right)=E_{c}/E_{m}$ with no filler particle–matrix interface present. Systems without the interface were studied in our previous work [89], so the value of $E_{0}$ can be considered a priori information for all relevant values of c and r. The case of $E_{s}=E_{p}$ corresponds to a proper increase in filler concentration, not an effective one:

(9) $$\frac{E_{c}\left(E_{s}=E_{p}\right)}{E_{m}}\approx 1+k_{1}\left(c+∆c\right),$$

For a cell containing N filler particles, one can then write the following:

(10) $$c+∆c=N\left(\frac{V_{p}}{V_{c}}+\frac{V_{s}}{V_{c}}\right),$$

In this work, the shape of the filler particles is that of a prolate ellipsoid with anisometry parameter, r. Using ellipsoid volume expressions, one can obtain the following:

(11) $$c+∆c=c{\left(1+\frac{h_{s}}{R}\right)}^{2}\left(1+\frac{h_{s}}{rR}\right),$$

Therefore, for a fixed value of $E_{s}$, the expression (7) for the cell modulus is as follows:

(12) $$\frac{E_{c}}{E_{m}}\approx 1+\frac{E_{0}-1}{c}c+\frac{E_{0}-1}{c}c\left({\left(1+\frac{h_{s}}{R}\right)}^{2}\left(1+\frac{h_{s}}{rR}\right)-1\right)S\left({\tilde{E}}_{s}\right),$$

An interface surrounding filler particles can be viewed as an additional material layer of the same shape as the particles. As such, the change in filler concentration can be considered from a geometric point of view. It is reasonable to assume that this volumetric effect can take the form of a scaling law. Based on the dependences presented in Figure 3c, an algebraic saturation function is chosen to describe the dependence of $E_{c}$ on $E_{s}$, and the scaling law is applied to it:

(13) $$\frac{E_{c}-E_{0}}{E_{m}}=\frac{∆E_{c}}{E_{m}}=\left(E_{0}-1\right)\left({\left(1+\frac{h_{s}}{R}\right)}^{2}\left(1+\frac{h_{s}}{rR}\right)-1\right)\frac{E_{s}/E_{m}-1}{\sqrt{k+{\left(E_{s}/E_{m}-1\right)}^{2}}},$$

The coefficient k can be calculated from the case of $E_{s}=E_{p}$. Calculations for spherical particles and oblate ellipsoidal particles can be performed in the same manner. Since the presence of the interface surrounding filler particles is considered as a volumetric effect, the comparison between cells containing spherical particles and cells containing anisometric particles should be made using the same value of $c_{eff}$ for both cases. If spherical filler particles are used as a baseline, then the value of $h_{s}$ for anisometric particles has to be adjusted in such comparisons. For example, the equivalent interface thickness for prolate ellipsoidal particles can be calculated using the following nonlinear equation:

(14) $$\left(1+\frac{h_{s}^{a}}{rR}\right){\left(1+\frac{h_{s}^{a}}{R}\right)}^{2}={\left(1+\frac{h_{s}^{i}}{R}\right)}^{3},$$

where $h_{s}^{a}$ is the thickness of the interface surrounding anisometric particles, and $h_{s}^{i}$ is the thickness of the interface surrounding spherical particles. When the cells containing spherical and ellipsoidal particles are compared, this equation is solved numerically, and the equivalent interface thickness value, $h_{s}^{a}$, is used for cells that contain ellipsoidal particles.

**Figure 4:**
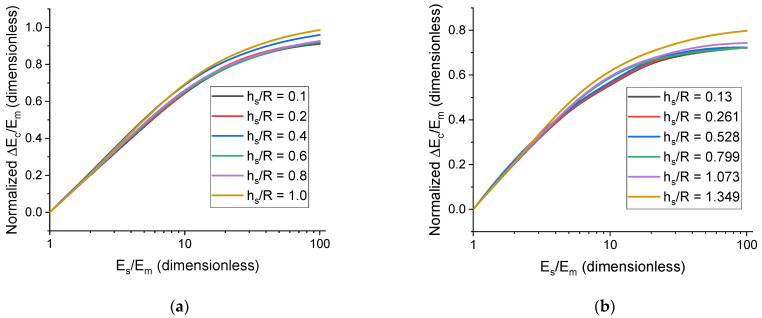
Normalized dependences of $∆E_{c}/E_{m}$ on ${\tilde{E}}_{s}$ (logarithmic scale) for different values of ${\tilde{h}}_{s}$ and filler volume concentration of 1% averaged by spatial orientation angles, θ and φ. (**a**) Corresponds to spherical particles. (**b**) Corresponds to particles with prolate ellipsoidal shape, r=3.

Another aspect of this problem that has not been addressed is the dependence of $E_{c}$ on the spatial orientation angles, θ and φ, for anisometric filler particles. Our calculations show that $E_{c}$ does not change significantly with φ for the values of filler concentration (c) and anisometry (r) considered here. The dependence of $E_{c}$ on θ is nonlinear, exhibits a minimum and saturation, and can noticeably affect the results, especially for cells containing prolate ellipsoidal particles. Therefore, to study the general trends, numerical solid angle averaging is performed for the cell modulus:

(15) $$\langle {\tilde{E}}_{c}\rangle =\frac{1}{4\pi }\int_{\Omega }{\tilde{E}}_{c}\sin\theta d\theta d\varphi ,$$

where Ω is the solid angle. Then, the master curves are calculated and studied using the average value of $E_{c}$. The brackets are omitted when talking about $\langle {\tilde{E}}_{c}\rangle$ for the sake of convenience. If the interface modulus, $E_{s}$, is lower than the matrix modulus, $E_{m}$, then the presence of the interface cannot be described as a volumetric effect; therefore, the case of ${\tilde{E}}_{s}<1$ is not considered in the scaling law (13). The dependences of $∆E_{c}/E_{m}$ on $E_{s}$ are shown in Figure 4. It can be seen that the curves calculated for different $h_{s}/R$ (see Figure 3a for spherical particles) values fall close to a master curve. Thus, the derived scaling law satisfactorily describes the effect of the particle shell for both spherical and anisometric particles.

**Figure 5:**
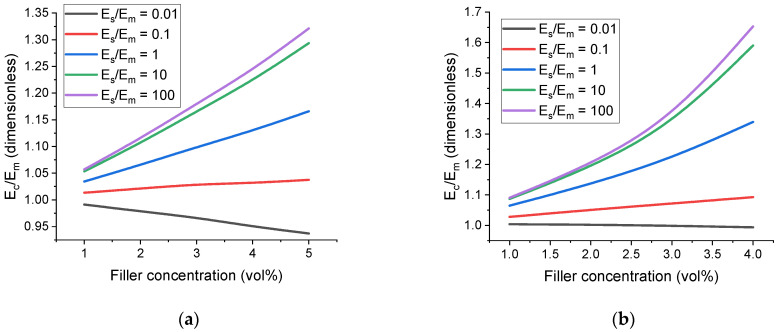
Dependences of ${\tilde{E}}_{c}$ on filler volume concentration, c, for different values of ${\tilde{E}}_{s}$. Interface thickness is ${\tilde{h}}_{s}=0.2$. (**a**) Corresponds to spherical particles. (**b**) Corresponds to particles with prolate ellipsoidal shape, r=3 and θ=90°.

Calculated dependences of ${\tilde{E}}_{c}$ on filler concentration are presented in Figure 5. A significant increase in the modulus of up to 20% due to the presence of a filler particle–matrix interface for a volume concentration of only 2% of highly anisometric particles was predicted in [104], and it is in agreement with the FEM simulation results presented in this work. As expected, the dependence is approximately linear at low filler concentrations. Deviations from the linear regime begin to be noticeable at an effective concentration of about 8% for spherical filler particles and about 5% for prolate ellipsoidal particles. The nonlinear behavior of ${\tilde{E}}_{c}$ for high values of ${\tilde{E}}_{s}$ corresponds to an increasing effective concentration. For example, if the filler concentration of oblate ellipsoidal particles is 4%, then the effective concentration for the case of ${\tilde{E}}_{s}=100$ and ${\tilde{h}}_{s}=0.2$ becomes 7.3%. It is clear that linear approximation for the dependence of ${\tilde{E}}_{c}$ on filler concentration no longer holds, and the expansion of the exponential function (5) has to contain quadratic terms:

(16) $$\frac{E_{c}}{E_{m}}\approx 1+k_{1}c+k_{2}c^{2}+\left(k_{1}∆c+2k_{2}c∆c+k_{2}∆c^{2}\right)\cdot S\left({\tilde{E}}_{s}\right),$$

This is an important factor to consider in future modeling.

**Figure 6:**
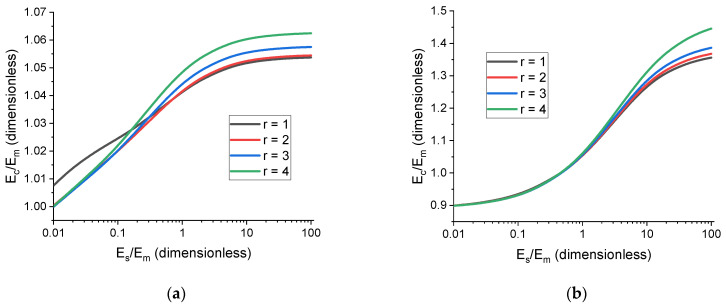
Dependences of ${\tilde{E}}_{c}$ on ${\tilde{E}}_{s}$ (logarithmic scale) for different values of prolate ellipsoidal particle anisometry parameter (r) averaged by spatial orientations, θ and φ. Volume concentration is c=1%. (**a**) Corresponds to ${\tilde{h}}_{s}=0.1$. (**b**) Corresponds to ${\tilde{h}}_{s}=1.0$.

Figure 6 demonstrates how filler anisometry affects the dependence of ${\tilde{E}}_{c}$ on ${\tilde{E}}_{s}$. Due to the difference in shape between the cases of r=1 and r>1, effective concentration ($c_{eff}$) was used as a fixed parameter to account for the presence of the filler particle–matrix interface and to compare values of ${\tilde{E}}_{c}$ for different values of r. It is evident that increasing filler anisometry leads to a more pronounced influence of the interface on the cell modulus, and the effect is nonlinear. Moreover, increasing $c_{eff}$ (or relative interface thickness) changes the ${\tilde{E}}_{c}\left({\tilde{E}}_{s}\right)$ dependence and leads to slower saturation with ${\tilde{E}}_{s}$ and higher cell modulus values.

## 4. Shell Around the Particle as a Stabilization Tool for Large Deformations

The movement of anisotropic filler particles in MAEs can cause large local deformations in soft polymer matrices. This can lead to large errors in numerical simulations even when the polymer medium is modeled as a hyperelastic material. An interface between the matrix and the filler particles can significantly improve solution convergence when $E_{m}<E_{s}<E_{p}$. The advantage of introducing such an interface into the model is twofold. On the one hand, it increases the scope of the modeling by allowing for simulations of filler particles covered by shells. On the other hand, it makes it possible to characterize the solution to the problem that does not contain an interface without solving it directly. This avoids the cases where convergence is not achieved. The neo-Hookean hyperelastic model is used in this work, and it is possible to use more complex hyperelastic models that can describe larger strains (such as the Mooney–Rivlin model); however, the neo-Hookean model requires less information about the material and, thus, less extensive experimental studies. This is especially relevant for designing new materials when a large number of options and components are considered. Shifting focus toward computer simulations during early design stages can help improve work efficiency. Therefore, circumventing the limitations of simpler mechanical models is important for practical applications of polymer material research. The dependence of any given characteristic, f, on the interface thickness ($h_{s}$) and Young’s modulus ($E_{s}$) can be calculated and approximated by a smooth function to obtain the value of f for $h_{s}\to 0$ or $E_{s}\to E_{m}$.

**Figure 7:**
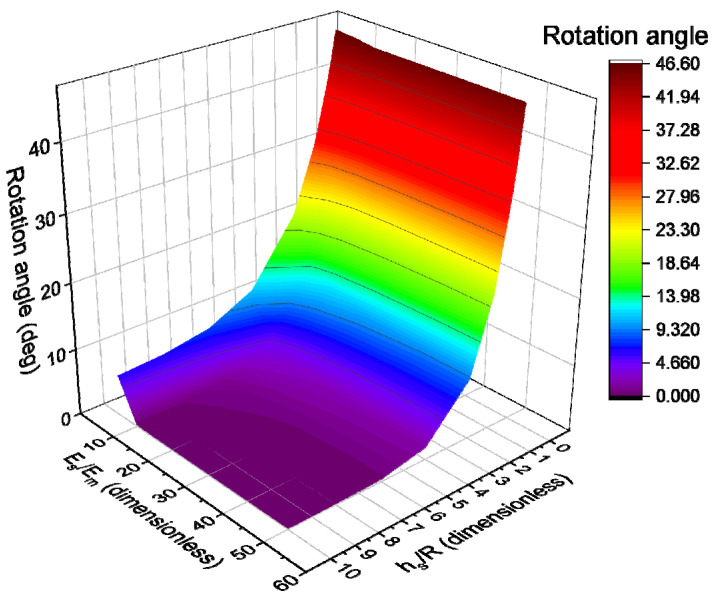
Dependence of the filler particle rotation angle (θ) in magnetic field, B=200 mT, on ${\tilde{h}}_{s}$ and ${\tilde{E}}_{s}$. The filler concentration volume is 1%, and the particle has a shape of a prolate ellipsoid with r=3.

This idea was tested using the rotation of a ferromagnetic ellipsoidal particle in uniform magnetic field. The angle of rotation, θ, was chosen as a relevant characteristic of the cell state (see Figure 7). The tests have shown that a simple exponential function can describe the behavior of $\theta \left(h_{s}\right)$ for a fixed value of external magnetic field, H, with a high degree of accuracy:

(17) $$\theta \left({\tilde{h}}_{s}\right)=\theta_{0}e^{-{\tilde{h}}_{s}/p}+\theta_{\infty },$$

Here, $\theta_{\infty }$ corresponds to the case of $E_{m}=E_{s}$ and $\theta_{0}+\theta_{\infty }=\theta \left(0\right)$.

The difference between the calculated $\theta \left(0\right)$ for different values of $E_{s}$ does not exceed 1.62%, thus demonstrating that the addition of an interface between filler particles and polymer matrix allows for calculating the characteristics of a solution of an interface-less problem without directly solving it. Additionally, those characteristics do not noticeably depend on the mechanical properties of the interface; therefore, the value of $E_{s}$ can be chosen arbitrarily to achieve faster convergence. The outlined method can be used to supplement simple hyperelastic models and analyze systems that would otherwise require more sophisticated mechanical descriptions.

## 5. Unit Cell Containing Several Filler Particles

**Figure 8:**
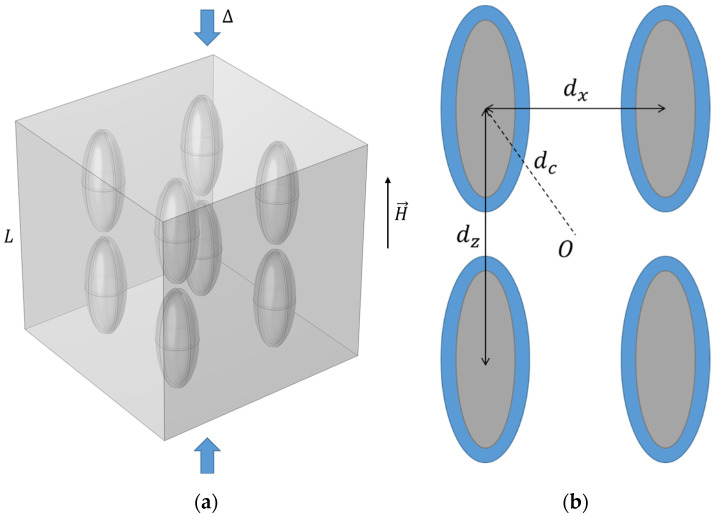
An example of a cell with n=8 ellipsoidal particles. (**a**) The general setup. (**b**) The distance between particles.

Filler particle arrangement and rearrangement play an important role in determining composite properties; therefore, next, a cell containing several particles arranged in different ways is studied. The directions of the vectors corresponding to the particle’s major axis, external mechanical load, and magnetic field are the most important factors when considering enhanced composite response; however, the distances between particles and particle aggregates in different directions can also influence the mechanical, dielectric, and magnetic properties of the material. For example, if particles form chain-like aggregates inside the composite, and the z-axis corresponds to the direction of those aggregates, distances in OX, OY, and OZ directions can be considered as mesoscopic structural parameters (see Figure 8).

Here, we consider cells containing n=8 and n=16 anisometric particles arranged in pre-determined ordered structures characterized by distances $d_{x}$, $d_{y}$, and $d_{z}$, as well as the spatial orientation angles, φ and θ. Assuming the mechanical load vector to have the same direction as z-axis, two reference configurations can be considered: lateral chains (θ=0°) and transverse chains (θ=90°). The number of particles in each chain, $n_{c}$, as well as the particle anisometry (r) and filler concentration (c), determines the distances $d_{x}$, $d_{y}$, and $d_{z}$. In this work, an idealized system is considered: each unit cell contains filler particles of the same shape, size, and spatial orientation with a particle–matrix interface of uniform thickness. While this idealization is significant, the general trends of the composite behavior can still be obtained using it, assuming that the filler structure was induced by external fields (i.e., magnetic field).

**Figure 9:**
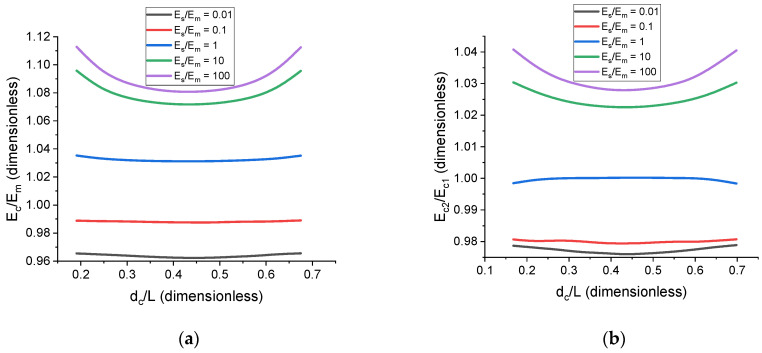
The effect of the interface on the dependence of the cell modulus, ${\tilde{E}}_{c}$, on inter-particle distance for prolate ellipsoidal particles, with n=8, r=3, and θ=90°. (**a**) Dependences of ${\tilde{E}}_{c}$ on ${\tilde{d}}_{c}$ for different values of ${\tilde{E}}_{s}$, ${\tilde{h}}_{s}=0.4$, and c=1%. (**b**) Dependence of the ratio $E_{c2}/E_{c1}$ on ${\tilde{d}}_{c}$ for different values of ${\tilde{E}}_{s}$ and c=1% with $E_{c1}$ corresponding to the case of ${\tilde{h}}_{s}=0.2$ and $E_{c2}$ corresponding to the case of ${\tilde{h}}_{s}=0.4$.

Changing the distances between particles affects the complexity of the overall 3D filler structure. For example, a chain-like structure can transform into a cluster-like structure if the inter-particle distance in a cell is either very small or very large. Therefore, quantifying the effect of the inter-particle distance on the mechanical properties of the cell can be of interest for designing composites. The simplest possible cell corresponding to the uniformly distributed chain-like structures is a cell with n=8 and $n_{c}=2$ (see Figure 8). In this simple and symmetric case, the distance from the particle center to the cell center, $d_{c}$, can serve as the main structural parameter for the sake of simplicity. Figure 9 demonstrates the general trends for the dependence of ${\tilde{E}}_{c}$ on normalized ${\tilde{d}}_{c}=d_{c}/L$ at c=1% and how the modulus and thickness of the surrounding particle–matrix interface can affect those dependences. At low concentrations, the effect is not pronounced: no interface being present ($E_{s}/E_{m}=1$) results in almost no dependence of the cell properties on $d_{c}$, while a stiff interface of medium thickness ($E_{s}/E_{m}=100$ and ${\tilde{h}}_{s}=0.2$) that corresponds to the effective increase in filler concentration from 1% to 1.73% results in the cluster-like structure increasing the cell modulus by 3% compared to the case of the chain-like structure. For a filler volume concentration of around 5%, this increase can reach 15%, which is noticeably more significant. Additionally, calculations show that, for uniform particle distribution (no clustering) and low filler volume concentration (i.e., c≤5%), single-particle cells and multi-particle cells are practically equivalent in terms of their mechanical response. Single-particle cells can therefore be used to estimate the general material behavior at low filler concentrations.

**Figure 10:**
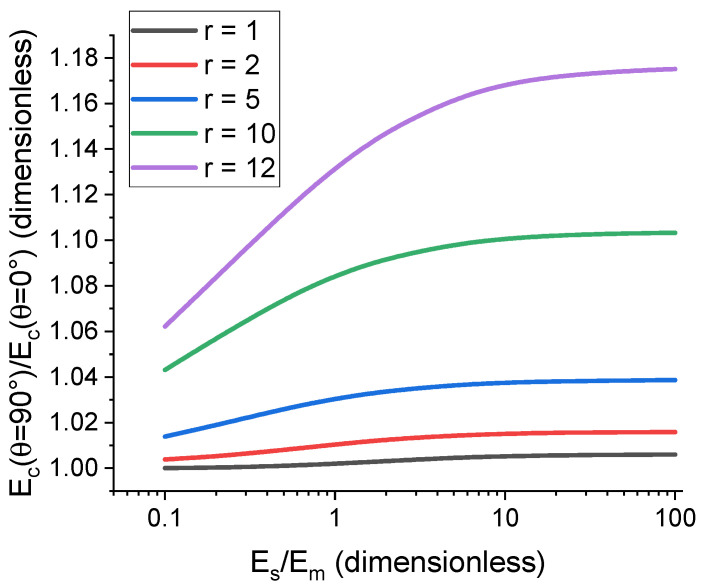
The effect the interface stiffness has on the difference in cell elastic modulus for different spatial orientations of the chain aggregates in a cell containing 16 oblate ellipsoidal particles. Each chain contains 4 particles, and the direction of the chain corresponds to θ. The filler volume concentration in this example is 1%, and the relative interface thickness, ${\tilde{h}}_{s},$ is 0.2.

To study how the mechanical properties of the cell differ between the cases of lateral and transverse chains and how these differences are affected by the particle–matrix interface, we consider cells with a higher degree of structural anisotropy containing n=16 particles and chains that have 4 particles each, or $n_{c}=4$. Cells containing transverse chains that align with the direction of the external mechanical load vector demonstrate noticeably higher cell modulus, a finding which is in agreement with published works on this topic [111]. Figure 10 shows the dependence of the ratio of $E_{c}\left(\theta =90{}^{\circ}\right)$ to $E_{c}\left(\theta =0{}^{\circ}\right)$ on the interface modulus for oblate ellipsoidal particles with different anisometry, r. The dependence becomes significantly more pronounced for high values of r even at low filler concentrations, thus further proving that anisometric fillers can enhance the composite properties more effectively than spherical fillers for low filling fractions. The cases of r≥10 simulate platelet filler particles that have attracted increasing attention from researchers in recent years [98,112]. Highly anisometric fillers (i.e., flake-like particles and nanotubes) are of particular interest for practical applications of polymer composite materials, as their influence on the material response is strong even at low filler concentration values.

**Figure 11:**
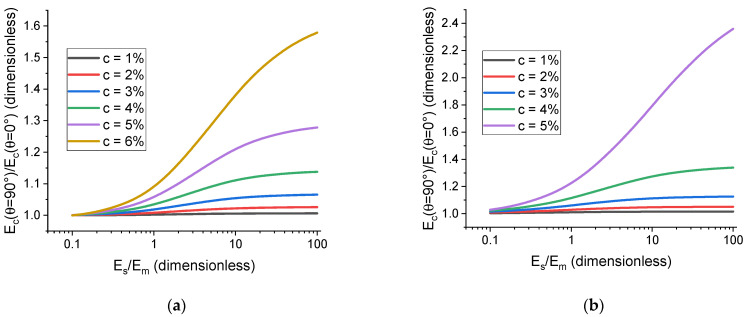
The effect the interface modulus has on the difference in cell elastic modulus for different spatial orientations of the chain aggregates in a cell containing 16 particles. Each chain contains 4 particles, and the direction of the chain corresponds to θ. The relative interface thickness, ${\tilde{h}}_{s},$ is 0.2. (**a**) Corresponds to spherical particles. (**b**) Corresponds to oblate ellipsoidal particles with anisometry r=2.

Figure 11 shows how these dependences change with increasing filler concentration, c. The curves are shaped like sigmoid functions, as is the case for single-particle cells, and increasing the concentration leads to saturation being achieved at higher values of ${\tilde{E}}_{s}$. This can be explained by interfaces surrounding different particles coming close to contact; therefore, the hydrodynamic interaction between the particles becomes stronger, and the difference between how the interface and the particle react to mechanical perturbations plays a bigger role in determining the overall mechanical response of the cell. In highly filled polymer composites used for engineering or biomedical applications, the volume fraction of the filler can reach 50% (or even higher values). Modeling such systems requires sufficiently accurate representation of particle interactions at small interparticle distances (including steric constraints relevant for anisometric filler particles) and is the subject of further consideration within the presented framework of the multi-particle unit cell approach.

The theoretical results presented here align well with the experimental observations [98]. As demonstrated in [98], the incorporation of magnetic particles can facilitate the fabrication of polymeric composites exhibiting anisotropic mechanical characteristics. Anisotropy is a direct consequence of the structuring of the magnetic filler in a magnetic field during the synthesis process. The chains of magnetic particles that are formed are fixed during the polymerization process. The elastic modulus of the resulting materials measured along the chain orientation, $E_{\|}$, is larger than the modulus $E_{⊥}$ measured in the perpendicular direction. It has been found that the anisotropy of mechanical properties is most clearly manifested in composites based on anisometric particles, including needle-shaped and flake-shaped particles [98], this fact being in agreement with simulations presented in this work. The highest values of the elasticity anisotropy coefficient, i.e., the ratio of the modulus values, $E_{\|}/E_{⊥}$, have been observed in MAEs containing flake-shaped iron microparticles, which are magnetized along the plane of the particles and rearranged with the particle planes parallel to the field, forming chains even at low volume fractions. The anisotropy increases with the filler concentration. Thus, the ratio of $E_{c}\left(\theta =90{}^{\circ}\right)$ to $E_{c}\left(\theta =0{}^{\circ}\right)$ calculated for different particle anisometries and different particle concentrations reflect the main tendencies observed for $E_{\|}/E_{⊥}$ of anisotropic MAEs in [98], providing a fundamental basis for their theoretical description.

## 6. Conclusions

In this study, the effect the interface between filler particles and polymer matrix has on local properties of filled polymer composites with low fraction of anisometric filler was modeled and analyzed. The interface was modeled as a shell with uniform thickness surrounding filler particles. The mechanical properties of the interface were set to be different from properties of the matrix and the particles. The local mechanical response of the composite was studied using a unit cell, i.e., a 3D cell containing one or several filler particles embedded in hyperelastic medium that represents the polymer matrix. The modeling was carried out using finite element simulations.

Unit cells containing a single particle with a particle–matrix interface surrounding it were analyzed. This case corresponds to very low filler concentrations; however, it is useful for understanding the general patterns of the local material behavior and how it can be changed via the introduction of the third phase represented by the matrix–particle interface. It was shown that while the extent to which the interface can increase or decrease the cell response is strictly limited by the quick saturation in the dependences of the cell elastic modulus on the interface elastic modulus and difficulties in synthesizing composites with thick interface surrounding filler particles, the change in the mechanical response of the composite containing 5% of highly anisometric filler particles of around 10–20% can be expected when using a moderately thick interface. It is worth noting that the presented model is dimensionless and scalable, and, thus, it can be used to describe both composites with micron-sized and nanometer-sized filler particles. It is possible to synthesize nanocomposites with sufficiently high interface layer thickness relative to the size of the nanoparticles, therefore increasing the influence of the interface on the material behavior and making it one of the primary aspects of the material design. Additionally, it was shown that the presence of the interface in the system can be modeled as an effective increase in filler concentration and a volumetric effect with specific scaling laws that can be obtained using classic filler reinforcement theory.

It was demonstrated that it is possible to use interface-based composite models as tools to stabilize the problems of mesoscopic filler restructuring with large deformations. Introducing interface that consists of one or several material layers around the filler particles creates additional damping in the model, and FEM calculations showed that the solution converges to the same point as interface thickness tends toward zero or interface modulus tends toward matrix modulus regardless of the initial interface characteristics or which quantity is chosen as a damping parameter. This can help solve mesoscopic continuum problems that do not allow for direct solutions.

Finally, it was considered how the particle-shape anisotropy and the presence of the particle–matrix interface could affect the anisotropy of the composite properties by analyzing unit cells containing an ordered structure of filler particles. It is widely known that field-sensitive polymer composites exhibit different mechanical responses in different directions due to filler restructuring in the presence of external field. According to the modeling results, introducing a moderately thick and sufficiently stiff particle–matrix interface can increase the difference between the mechanical response of the composite in different directions by around 20% for composites with a low filler concentration. It is important to note that, for high filler fractions, this effect may not be as pronounced due to the limitations the interface surrounding filler particles puts on their movement.

The areas of future work on mesoscopic modeling of polymer composites and the influence of the filler particle–matrix interface on their properties include addressing the shortcomings of the approach presented in this paper, namely describing the softening of the material when the elastic modulus of the interface is lower than that of the polymer matrix, calculating the response of the cells containing randomly distributed filler particles, accounting for polydispersity of the filler, and studying how the presence of the interface affects interparticle interactions.

The considerations and results presented in this article show that the introduction of the sufficiently thick particle–matrix interface into the filled polymer composites can allow for further fine-tuning of their mechanical properties, in addition to controlling filler concentration and filler shape, and can be used as a useful tool for designing new smart materials in the future.

## Data Availability

The original contributions presented in this study are included in the article. Further inquiries can be directed to the corresponding author.
